# Immunomodulatory and Antitumoral Activity of Gold Nanoparticles Synthesized by Red Algae Aqueous Extracts

**DOI:** 10.3390/md20030182

**Published:** 2022-02-28

**Authors:** Noelia González-Ballesteros, Lara Diego-González, Mariano Lastra-Valdor, Maria Grimaldi, Antonella Cavazza, Franca Bigi, M. Carmen Rodríguez-Argüelles, Rosana Simón-Vázquez

**Affiliations:** 1 CINBIO, Departamento de Química Inorgánica, Universidade de Vigo, 36310 Vigo, Spain; noeliagb@uvigo.es; 2 CINBIO, Immunology Group, Universidade de Vigo, 36310 Vigo, Spain; ldiego@uvigo.es (L.D.-G.); rosana.simon@uvigo.es (R.S.-V.); 3 Instituto de Investigación Sanitaria Galicia Sur, Hospital Alvaro Cunqueiro, 36312 Vigo, Spain; 4 CIM, Universidade de Vigo, 36331 Vigo, Spain; mlastra@uvigo.es; 5 Dipartimento Scienze Chimiche, della Vita e della Sostenibilità Ambientale, Università di Parma, 43124 Parma, Italy; maria.grimaldi@unipr.it (M.G.); antonella.cavazza@unipr.it (A.C.); franca.bigi@unipr.it (F.B.); 6 Institute of Materials for Electronics and Magnetism, National Research Council, 43124 Parma, Italy

**Keywords:** green synthesis, *Chondrus crispus*, *Gelidium corneum*, *Porphyra linearis*, gold nanoparticles, antioxidant activity, antitumoral activity, anti-inflammatory activity

## Abstract

This study reports on the green and cost-efficient synthesis of gold nanoparticles from three different red algae extracts. The nanoparticles synthesized were fully characterized by UV-Vis spectroscopy, HRTEM, and Z-potential. Relevant components occurring in the extracts, such as polysaccharides or phenolic content, were assessed by analytical techniques such as spectrophotometric assays and liquid chromatography. Finally, the antioxidant, antitumoral, and anti-inflammatory potential of both the extracts and the gold nanoparticles synthesized were analyzed in order to determine a possible synergistic effect on the nanoparticles. The results obtained confirmed the obtainment of gold nanoparticles with significant potential as immunotherapeutic agents. The therapeutic potential of these nanoparticles could be higher than that of inert gold nanoparticles loaded with bioactive molecules since the former would allow for higher accumulation into the targeted tissue.

## 1. Introduction

Among the large number of nanomaterials available, gold nanoparticles (AuNPs) have been attracting enormous attention on account of their outstanding physico-chemical properties, which make them suitable for application in many fields such as the pharmaceutical and food industry [1,2,3]. AuNPs are of particular interest in the field of nanomedicine, since the study of their properties has revealed that they could be valuable tools for the diagnosis and treatment of cancer [4]. Moreover, their antimicrobial, antiviral, and antioxidant capacity has also been investigated [5]. Recently, some researchers have also studied their possible employment in the treatment of inflammatory disease drawing on the different mechanisms of anti-inflammatory response linked to AuNPs, such as their capacity to reduce the production of reactive oxygen species (ROS) or decreasing lipopolysaccharide (LPS) induced cytokine production [6,7]. Cytokines are signaling proteins that participate in the inflammatory and immune response and mediate many physiological and pathological processes, such as aging or infection. Depending on the profile of cytokines induced by a stimulus, a different inflammatory or immune response is triggered in the organism [8]. The use of cytokines or molecules that can modify the cytokine profile is a relevant area of research in immunotherapy [9]. Immunotherapy is a very promising therapeutic approach, alone or in combination with other therapies, in the treatment of many chronic diseases where the immune system is overreacting (autoimmunity and inflammation) or suppressed (immunosuppression and immunodeficiency). In fact, tuning the immune system to treat chronic inflammatory diseases or to fight cancer has already shown a great potential [10]. Similarly, the efficacy of an adjuvant in the development of preventive or therapeutic vaccines is mediated by its capacity to activate the antigen presenting cells through the induction of cytokine release, among other mechanisms [11].

Besides, the production of ROS plays a relevant part in the activation of innate and adaptive immunity. For that reason, ROS modulation is relevant to immune-mediated responses, such as antitumor immunity [12]. In fact, the antitumoral effect of antioxidant molecules from algae has pointed at their relevant therapeutic potential, not only to tackle some tumoral diseases but also to protect non-tumoral cells. However, some factors, such as the dose or the potential interaction with some antineoplastic drugs, should be carefully evaluated [13].

As happens in the case of many other therapeutic approaches, the use of a nanocarrier can improve the release and reduce the off-target effects of the immunomodulatory drug. Additionally, if the nanocarrier itself possesses immunomodulatory activity, instead of being a passive carrier, its therapeutic efficiency could be improved and side-effects reduced [14].

Hence, the use of nanoparticles (NPs) with immunomodulatory effects synthesized using green methods is a novel and underexplored area of research with a great potential in immunotherapy [15]. Following the principles of the green chemistry, we aim to produce safer and more biocompatible NPs, reducing the use of hazardous chemicals and solvents, avoiding or reducing the generation of wastes, while using a mild condition, i.e., atmospheric pressure and room temperature, when possible [16,17]. These principles also include the use of raw material or feedstock [18]. In this regard, following our experience with brown [19,20] and green algae [21,22], this study focused on the green synthesis of AuNPs mediated by red seaweeds. Red seaweed or Rhodophytes are one of the three main phyla of algae. They are a diverse group that is estimated to contain around 7000 species. Due to the wide diversity of biomolecules that they present, they are promising organisms when it comes to the development of new biologically active compounds, which could be employed to devise novel nutraceutical, cosmeceutical, and pharmaceutical products [23]. The main compounds studied are polysaccharides, phenols, pigments, and fatty acids. The biological activities that most of them have shown—particularly their antimicrobial, antiviral, antitumor, antioxidant, antidiabetic, and analgesic effects—make them a valuable resource [24,25,26]. Among these effects, the anti-inflammatory activity observed in different polysaccharide fractions extracted from red seaweed has been recently highlighted [27,28].

In this study, three red seaweed were selected, namely, *Chondrus crispus* Stackhouse, *Gelidium corneum* (Hudson) J.V. Lamouroux, and *Porphyra linearis* Greville. These species were selected for their abundance and increased interest of their compounds in the treatment of cancer, infectious disease, and inflammation [29]. *Chondrus crispus* (hereafter CC) is a temperate latitude red macroalgae from the Family Gigartinaceae that is found abundantly on the Atlantic coasts of Europe and North America. It is a source of carrageenan, a polysaccharide widely used in the food, cosmetic, and healthcare industries as a thickening or stabilizing agent [30]. Recent studies have shown the potential health benefits of bioactive molecules extracted from this seaweed, particularly with regards their immune-stimulant effect [31,32]. Furthermore, the antitumoral potential of an aqueous CC extract rich in carrageenan have been described to be effective against four human cancer cell lines [33], while another study revealed that flavonoids, phenols, and tannins from CC methanolic extract exhibit significant antioxidant, anti-inflammatory, and cytotoxic properties in several human cancer cell lines [34]. *Gelidium corneum* (hereafter GC), formerly *G. sequispedale*, is of significant commercial interest since it is used to produce agar-agar [35]. In recent years, it has been studied in relation to its use in the production of bio-ethanol and antimicrobial biofilms for food packaging [36,37,38]. However, the number of studies dealing with the study of the biological properties of this specie is scarce. Recently, it was reported the immunomodulatory and antioxidant activities of sulfated polysaccharides extracted from GC [39]. *Porphyra linearis* (hereafter PL) is a common winter red macroalga in the Order Bangiales, widely spread in the high littoral and zones of northern Atlantic and Mediterranean coasts [40,41]. The genus *Porphyra* includes important red algal species that are cultivated and/or harvested for human consumption because of their high content of proteins (25–50%), vitamins, and fiber [42]. To the best of our knowledge, this is the first report on the biological activity of this *Porphyra* specie and its extracts. Other *Porphyra* species and their components have been reported to present notorious pharmacological potential. For instance, the sulphated polysaccharides extracted from *Porphyra umbilicalis* showed immunomodulatory and antioxidant activities [39], while other authors reported the antitumor activity of the polysaccharide fraction extracted from *Porphyra Haitanensis* [43].

These three kinds of seaweed were used for the preparation of aqueous extracts that were then employed to produce AuNPs. The antioxidant, antitumoral, and anti-inflammatory potential of the AuNPs were analyzed and compared with that of the extracts.

## 2. Results and Discussion

### 2.1. Synthesis and Characterization of AuNPs

In this study the use of three red macroalgae for the reduction of Au(III) to Au(0) was examined, CC, GC, and PL being the extracts employed for the synthesis. With the aim of obtaining homogeneous and narrow size distributions of the NPs, several reaction conditions were tested, by modifying extract concentration, gold concentration, temperature (T), and time (t). The reactions were monitored in the first place by color change and UV-Vis spectroscopic analysis. After a first evaluation by TEM, the best reaction conditions for each species are shown in Table 1. The full characterization of the NPs was made on samples obtained under these conditions.

**Figure 1:**
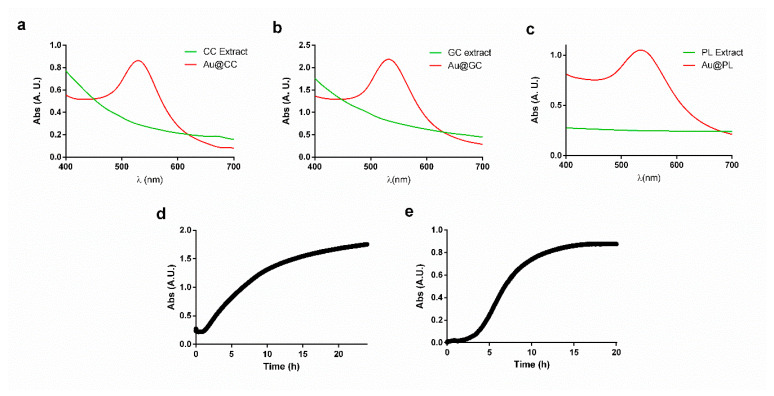
UV-Visible spectra analysis of (**a**) CC extract, (**b**) GC extract, and (**c**) PL extract before and after the synthesis of AuNPs; (**d**,**e**) time course spectra measurements of Au@GC and Au@PL, respectively.

In the case of Au@CC, after 48 h of reaction at room temperature a change in color, from yellow to red, was observed. UV-Vis spectra analysis showed the presence of the surface plasmon resonance (SPR) band of gold with maximum wavelength (λ_max_) at 529 nm (Figure 1a). Castro et al. reported on the synthesis of AuNPs using dried powder of *C. crispus* biomass at different pHs and obtained different λ_max_, with values ranging from 540 nm to 800 nm [44]. Figure 1b shows the UV-Vis spectra obtained for GC extract before and after the synthesis of NPs. The appearance of the SPR band with a λ_max_ at 532 nm can be observed. Kumar et al. reported on the synthesis of gold nanocubes employing dry powder of *Gelidium amansii* biomass, and, although they synthesized NPs with different shape and capping, they obtained a SPR band at a similar wavelength [45]. Several assays were needed to optimize the reaction conditions for the synthesis of AuNPs led by the aqueous extract of PL. When adding different concentrations of HAuCl_4_ to the concentrated extract, both color change (from pale pink to grey) and the formation of a dark precipitate were observed. Interestingly, when decreasing the concentration of the extract, the quantity of precipitate formed diminished, and the color of the solution shifted towards blue and, finally, purple. UV-Vis spectra analysis (Figure 1c) showed the presence of the SPR band with λ_max_ at 531 nm.

To obtain more information regarding the time needed for the reaction to conclude, UV-Vis spectra at the maximum of absorbance wavelength were recorded every ten seconds. In the case of Au@CC, it was impossible to obtain the spectrum due to the long time the reaction needs (48 h). The spectrum obtained for Au@GC is shown in Figure 1d. It can be clearly seen that the reaction is divided into three stages. The first one corresponds to the activation process between 0 and 2.5 h. Then, between 2.5 and 10 h, an increase in absorbance was observed. It was at this stage when color changed and the nucleation process might have taken place. After that, the reaction slowed down, and no further variation in color was observed. The reaction was stopped after 24 h. In the case of Au@PL, as shown in Figure 1e, the reaction was faster than in the case of Au@GC. The activation process occurred in the first 2 h. Next, between 2 and 10 h, an increase in absorbance was registered. After that, the reaction slowed down, but it did not stabilize until after 20 h.

Z potential measurements were conducted in order to analyze the surface charge of the colloidal suspension and evaluate the stability of the NPs. The Z potential values obtained were −18.8 ± 0.8 mV for Au@CC, −12.4 ± 2.3 mV for Au@GC, and −2.8 ± 1.4 mV for Au@PL. The values obtained revealed that the NPs synthesized formed a colloidal suspension where the particles carried a negative electrostatic surface charge. This negative charge could be due to the presence of polysaccharides in the extract. Regarding stability, Au@PL was the least stable sample obtained, and this might be due to the dilution of the extract, which also affects the capping agents present. Au@CC and Au@GC have been confirmed to possess good long-term stability (>3 months) when samples are preserved at 4 °C.

**Figure 2:**
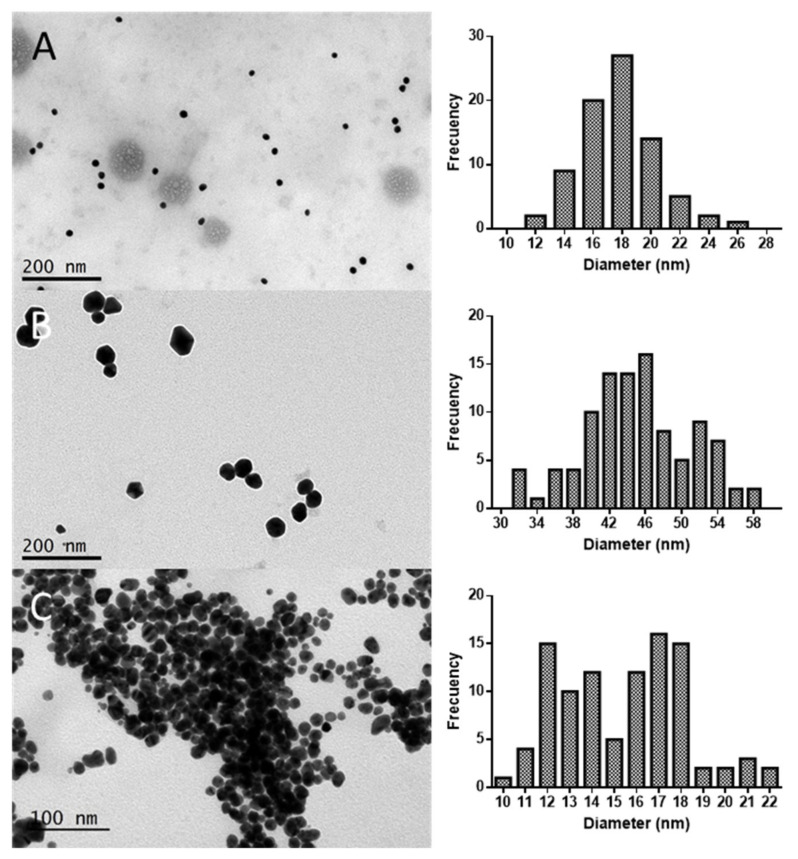
Transmission Electron Microscopy images of (**A**) Au@CC, (**B**) Au@GC, and (**C**) Au@PL, along with the size distribution histograms calculated.

TEM images are shown in Figure 2, along with the size distribution histograms calculated for each sample. As it can be observed in the figure, Au@CC and Au@PL were spherical, while the formation of polyhedral NPs can be observed in the case of Au@GC.

As shown in Figure 2A, Au@CC presented a mean diameter of 16.9 ± 2.5 nm. In the work of Castro et al., where they reported on the synthesis using dried powder of *C. crispus* biomass, a mixture of sizes and shapes was obtained, with spherical, triangular, and hexagonal NPs, with diameters between 30 and 200 nm [44]. The largest NPs were obtained with GC extract, as shown in Figure 2B, with a mean diameter of 44.2 ± 6.1 nm. The NPs obtained with PL extract were similar in size to those obtained with CC, as shown in Figure 2C. However, it can be noted that in this sample the NPs are more aggregated than in the case of Au@CC and Au@GC. The reason for this might be the fact that the extract was diluted for the synthesis of the NPs. This is in accordance with the Z potential value obtained, which showed that Au@PL were the least stable NPs synthesized.

**Figure 3:**
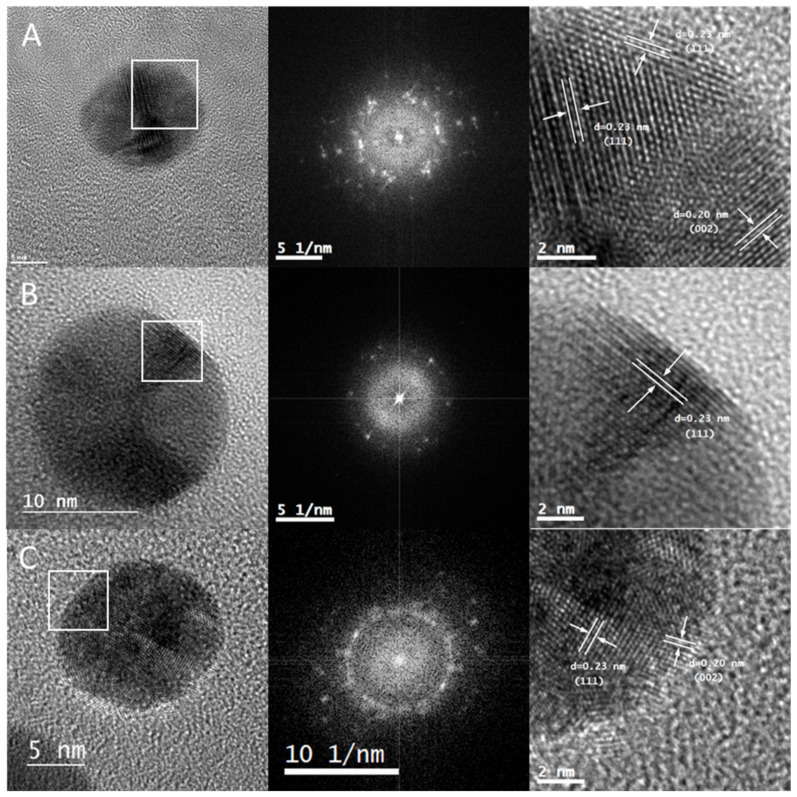
High-Resolution Transmission Electron Microscopy images of the selected AuNPs, along with the corresponding Fourier Transform, d-spacing, and Miller index calculated for the marked area of (**A**) Au@CC, (**B**) Au@GC, and (**C**) Au@PL.

The crystalline nature of the AuNPs synthesized was analyzed by means of HRTEM image acquisition. Figure 3A–C shows the images of Au@CC, Au@GC, and Au@PL, respectively, along with the corresponding Fourier Transform (FT) analysis. The acquisition of HRTEM images in these samples was challenging since an organic layer was quickly deposited on the surface of the NPs due to the nature of the extracts. It can be observed that the particles selected as examples display internal complex contrast. The FT confirmed that the NPs analyzed were polycrystalline, with the images showing clusters of spots instead of a uniform pattern. This seems to be a general trend in all the samples.

The d-spacing of the crystalline structure of the selected NPs was calculated in the marked area. In the three samples the preferential d-spacing of 0.23 nm was measured, corresponding to Miller index (111). In the case of Au@CC and Au@GC a d-spacing of 0.20 nm was also measured and was assigned to Miller index (002). All the results are consistent with a face-centered cubic crystalline structure for gold.

**Figure 4:**
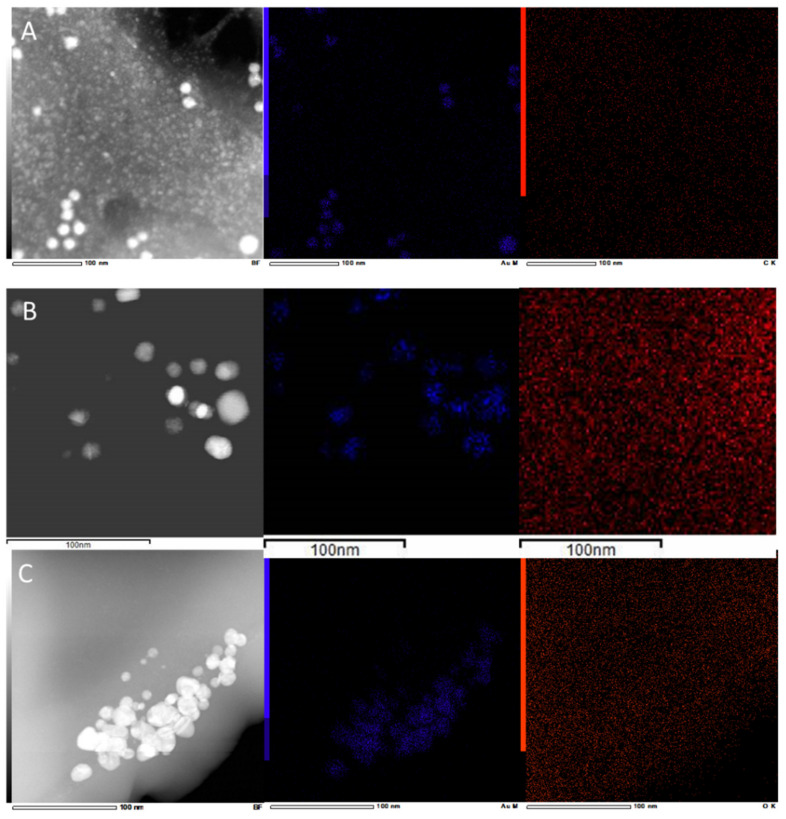
Dark field Scanning Transmission Electron Microscopy image, gold, and carbon mapping of (**A**) Au@CC, (**B**) Au@GC, and (**C**) Au@PL.

Dark Field-STEM images were also acquired (Figure 4). The contrast of the images depends on the atomic number of the atoms present in the samples, so AuNPs appear with a brighter contrast surrounded by a mass with a darker contrast. This can be seen more clearly in Figure 4A,B, corresponding to the samples of Au@CC and Au@GC, where the extract is more concentrated. In the case of Au@PL, this organic mass is not clearly observed, due to, on the one hand, the dilution of the sample for the synthesis of the NPs and, on the other, the centrifugation process that was performed for the TEM grid sample preparation.

**Figure 5:**
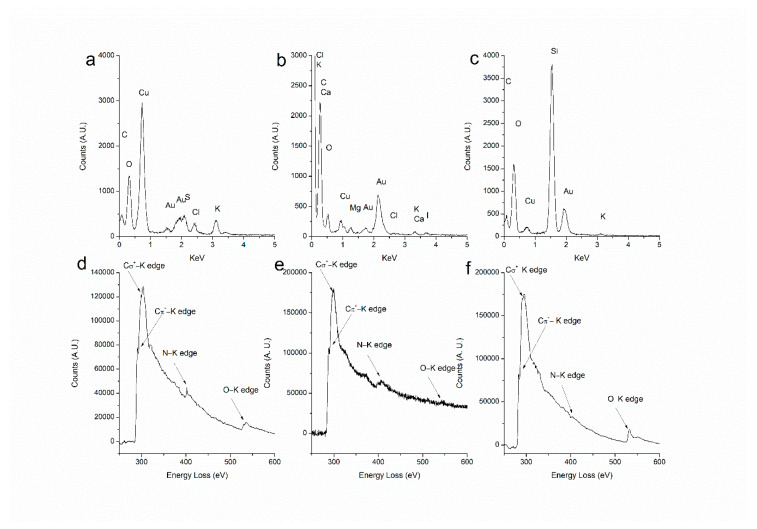
(**a**–**c**) Energy Dispersive X-ray Analysis of Au@CC, Au@GC and Au@PL, respectively. (**d**–**f**) Electron Energy Loss Spectroscopy Analysis of Au@CC, Au@GC, and Au@PL, respectively.

Energy Dispersive X-ray Analysis was performed (Figure 5). Apart from confirming the presence of gold, other elements present in the seaweed were also detected. In the case of Au@CC, the spectrum showed the presence of C, Cl, K, S, and O. As regards the sample of Au@GC, C, Ca, Cl, K, I, Mg, and O were identified. The elements detected in the samples had been previously identified as components of *C. crispus* [46] and *G. corneum* [47]. In the case of the NPs synthesized by PL extract, the EDX Analysis showed the presence of C, K, and O. In this sample, the effect of the dilution can be clearly seen in the results obtained, since only major components appear in the spectrum. A previous study had reported on a high content of potassium in other *Porphyra* [48]. The copper signal in the spectrum might be attributed to the copper grids but also to the composition of the macroalgae since it appears as a trace element [46,47,48]. Figure 4 represents the elemental mappings obtained from the EDX spectra by selecting carbon and gold. In all cases, it was confirmed that carbon is surrounding the NPs, while gold appears to be concentrated in the NPs and does not appear within the extract, which would confirm the full reduction of HAuCl_4_.

Electron Energy Loss Spectroscopy was also performed for the characterization of the organic extract since this technique is more recommendable for the analysis of lower atomic number elements than EDX. Figure 5 shows the EELS spectra of the three samples. In all of them, the edges of carbon (284 KeV), nitrogen (401 KeV), and oxygen (532 KeV) can be observed, although the signal of N in Au@PL is less intense, which could be accounted for by the lower concentration of extract employed for the synthesis. All these assays corroborate that the mass surrounding the NPs is organic. It can be concluded that the AuNPs are embedded within the algae extract, and they act both as a reducing and a stabilizing agent that prevents the particles from aggregating and precipitating.

Size Exclusion Chromatography has been demonstrated to be an analytical technique that allows for a simple and rapid characterization of the carbohydrate fraction occurring in different seaweed samples, in terms of molecular mass distribution [49]. Red algae are known to contain large amounts of polysaccharides belonging to the family of sulfated galactans, which includes agar, agarose, and carrageenans, to the family of sulfated mannans, or to the family of neutral xylans [50]. Each species is characterized by a different molecular distribution, influenced by many parameters such as the life cycle stage, season, environmental conditions of growth, and biotic interactions [51].

**Figure 6:**
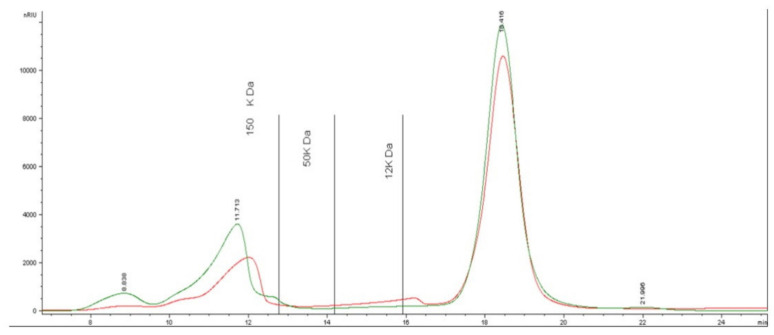
Chromatographic profile of PL extract: green line—aqueous extract; red line—extract after the formation of NPs.

In this study, the evaluation of the chromatograms recorded permitted to observe different profiles for each seaweed sample. An example, related to the PL aqueous extract, is provided in Figure 6, where the chromatographic profile shows multiple bands eluting in different regions. The comparison between the retention times of the recorded signals and those of calibration standards (displayed in the graph as vertical lines) provides information about the molecular weight (MW) of the different polysaccharide fractions. In detail, for PL, 69% of the total amount is constituted by molecules with MW below 12 kDa, whereas 31% belongs to MW higher than 150 kDa. In this region, two different eluting bands are evidenced, a small one appearing at about 8.8 min, and a more pronounced one eluting at about 11.7 min. The average MW calculated was about 55 kDa.

CC showed a similar qualitative profile, although characterized by a definitely higher percentage of the fraction above 150 kDa, represented by a large and broad peak, which accounts for about 48% of the total amount.

As for GC, the distribution of MWs was quite different, with 61% of molecules with MW higher than 150 kDa, and three small fractions of about 10–15% each, eluting in the different regions characterized by MWs 150–50 kDa, 50–12 kDa, and <12 kDa, respectively. Therefore, the average MW was about 76 kDa for CC and 107 kDa for GC.

After the formation of the NPs, slight changes could be perceived in different regions of the chromatographic profiles, as shown by the red line in Figure 6. The differences observed can be related to the implication of polysaccharides in the formation and the stabilization of the NPs, as already reported in previous studies [21]. It can be noted that the first small band eluting at about 8.8 min disappeared completely, accompanied by a sensible difference in the area of the other two main bands, with a decrease of 35% and 7%, respectively. Besides, the peak eluting at 11.7 min slightly shifted towards longer retention times.

As a consequence of the formation of NPs, the average MW calculated was found to decrease in PL, from 55 to 45 kDa. In the other seaweed examined, a different trend was recorded, with major modifications related to the last eluted bands, leading to an increase in the average MW from 85 to 92 kDa in CC and from 107 to 117 kDa in GC.

The recorded data suggest that each seaweed species has its own distribution of the carbohydrate chains, and the behavior of the fractions at different MWs during nanoparticle formation is probably linked to the initial carbohydrate chains occurring in the extract, depending on the available hydroxyl groups that participate in the reaction. In any case, there seems to be a stronger involvement of the smaller molecules in the reaction.

**Figure 7:**
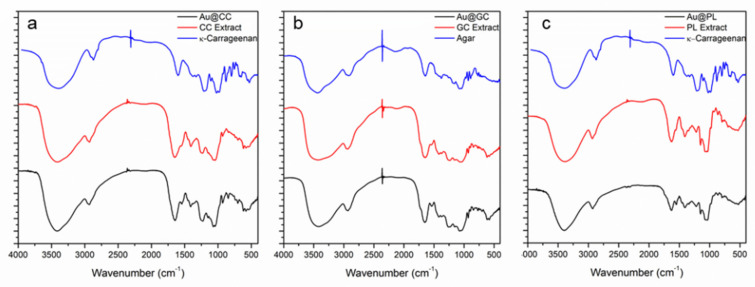
Fourier Transform infrared spectra of the *C. crispus* (**a**), *G. corneum* (**b**), *and P. linearis* (**c**) extracts before (black line) and after the synthesis of AuNPs (red line) compared with commercial polysaccharides (blue line).

Fourier Transform Infrared Spectral Analysis of the seaweed extracts before and after the synthesis of AuNPs was performed. After confirming the presence of polysaccharides in the extracts, their FTIR spectra were compared with those of commercial polysaccharides. In the case of CC and PL, carrageenan was chosen (Figure 7a,c) [30], while in the case of GC, the commercial polysaccharide selected for the comparison was agar, since several studies report on the extraction of this polysaccharide from different species of *Gelidium* (Figure 7b) [52].

Vibration bands were assigned following previous studies for *C. crispus* [44,53,54,55,56] and *G. corneum* [53,57,58,59]. In the case of *P. linearis,* there were no previous studies on the FTIR analysis of this seaweed or their extracts, so vibration bands were assigned following previous studies on other species of *Porphyra* [53,60,61].

Five main regions can be distinguished as regards the distribution of the bands in all the spectra. First, there is an intense and broad absorbance region at approximately 3400 cm^−1^, which can correspond either to the O-H stretching vibration of hydroxyl groups in alcohols or to the N-H stretching vibrations in amides and amines. Second, the band that appears between 2934 and 2920 cm^−1^ can be attributed to C-H stretching vibration modes of the hydrocarbon chains. Third, carboxylate groups show two bands: an intense asymmetrical stretching band at around 1650 cm^−1^ and a weaker symmetrical stretching band at 1400 cm^−1^. Fourth, bands between 1200 and 970 cm^−1^ are typically attributed to C-C and C-O stretching bonds and glycosidic C-O-C vibrations, common to all polysaccharides. Finally, the signals attributed to the sugar ring and glycosidic bond C-O stretching vibrations appear at 1050 cm^−1^. The presence of sulfate groups in the polysaccharide structure can be confirmed by the appearance of C-O-S bending vibration at 800 cm^−1^ as well as S-O stretching vibration at 1250 cm^−1^, attributed to sulfated esters. Some works have proposed that the intensity of this band is related to the degree of sulfonation of the polysaccharide [54].

When comparing the spectra for commercial carrageenan with the ones for CC and PL extracts, it could be observed that the main bands of carrageenan appeared in the samples with some shifts. The band that appears in carrageenan at 3438 cm^−1^ shifted toward lower wavenumbers, 3421 for CC and 3392 cm^−1^ for PL. As regards the band at 1652 cm^−1^, in the case of CC, there was no shift in the wavenumber, but the appearance of a shoulder was observed. In the case of PL, there was a shift to lower wavenumbers (1622 cm^−1^), and the appearance of a shoulder was observed. Major differences were observed in the region between 1400–700 cm^−1^, with differences in the intensity and position of the bands. The peak at 1424 cm^−1^ shifted to 1407 and 1408 cm^−1^ in CC and PL extracts, respectively. The intense band at 1260 cm^−1^ shifted to lower wavenumbers, 1237 and 1221 cm^−1^ in CC and PL extracts, respectively. This might be attributed to the low selectivity of aqueous extraction where other biomolecules from the algae, such as proteins or polyphenols, are also extracted. So, the presence of these molecules in association with carrageenan could account for the mentioned shift.

In the case of CC extract, when comparing the spectra with the one of the AuNPs synthesized, it was observed that the peak corresponding to carbonyl stretching at 1652 cm^−1^ shifted to lower wavelengths (1649 cm^−1^), while the peak at 1407 cm^−1^ shifted to higher wavelengths (1412 cm^−1^). Additionally, the bands at 1237 and 1072 cm^−1^ shifted towards lower wavelengths (1228 and 1069 cm^−1^). In the case of PL extract, when compared with Au@PL, some shifts in the bands were observed. Firstly, in the peak at 3390 cm^−1^ a difference in width and intensity was seen. The major changes were noted in the peaks at ~1600 and ~1400 cm^−1^, assigned to the asymmetrical and symmetrical stretching bands of carbonyl groups that shifted towards higher wavelengths, from 1622 to 1630 and from 1408 to 1411 cm^−1^. Lastly, in the bands at 1200 and 1050 cm^−1^, a reduction in intensity was observed. The results obtained for both seaweed are in accordance with other works that have proposed that the carbonyl group from proteins is able to bind metals, and, as a result, they could most possibly cap AuNPs and, thus, prevent agglomeration [20,21,62]. The modifications observed in the region of 1200–1000 cm^−1^ could indicate that sulfonic groups from polysaccharides are involved in metal binding. In this sense, carrageenan, due to the amount of sulfur it possesses, is able to bind gold and work as a capping and stabilizing agent of NPs [44,63].

Regarding the FTIR spectra of agar and GC extract (Figure 7b), a similar pattern in the bands can be observed. However, there are some shifts that can be attributed to the extraction of other biomolecules, such as polyphenols and proteins. Furthermore, the main difference is observed in the band at 1239 cm^−1^, assigned to SO_3_^−^ stretching mainly present in sulfonic acids of polysaccharides that do not appear in the commercial agar. This could be explained by the fact that agar consists of two polysaccharides, agarose, and agaropectin. Agarose contains 1, 3 linked D-galactose and 1, 4 linked 3, 6 anhydro L-galactose units, with a low percentage of hydroxyls being sulfated. The structure of agaropectin is more complex. Apart from D-galactose and 3, 6 anhydro galactose units, it also contains D-gluconic acid, pyruvic acid, and a much higher proportion of sulfate ester groups [64]. Therefore, it could be argued that in the extract there was a higher content of sulfate esters than in the commercial agar. In the GC extract spectrum, a band appeared at 3423 cm^−1^, which may correspond to NH or OH stretching vibrations of amino or hydroxyl groups. The CH stretch band was observed at 2934 cm^−1^. As previously indicated, carbonyl groups account for two bands that, in this case, appeared at 1652 cm^−1^ and 1418 cm^−1^. C-OH vibrations of primary alcohol groups are related to the band at 1069 cm^−1^, and the band at 1239 cm^−1^, as previously mentioned, could be associated with—SO_3_^−^ stretching.

The comparison of these bands with the ones obtained for Au@GC revealed only minor shifts regarding position and intensity, except in the case of the band at 1239 cm^−1^, where a major shift to a higher wavelength (1259 cm^−1^) was observed. This could suggest that polysaccharides probably bind gold through the sulfur atoms and can act as a capping and stabilizing agent of NPs.

### 2.2. Antioxidant Activity

**Figure 8:**
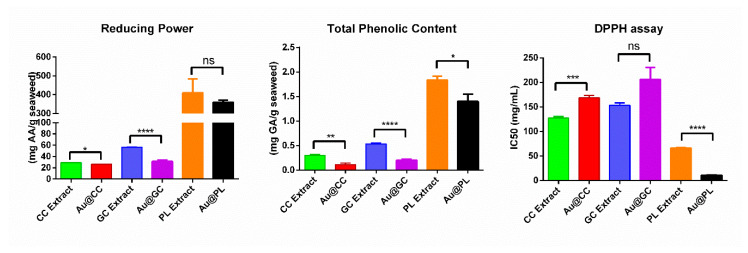
Graph bars of the reducing power, total phenolic content, and DPPH scavenging activity of CC, GC, and PL extracts and their AuNPs. ns *p* > 0.05, * *p* ≤ 0.05, ** *p* ≤ 0.01, *** *p* ≤ 0.001, **** *p* ≤ 0.0001.

The results from the assays performed to determine the reducing power, total phenolic content, and DPPH scavenging activity are collected in Figure 8. Regarding the results for the reducing power, a wide range of values can be observed in the three different species. PL possesses the highest value among them, with a reducing power comparable to that of the brown seaweed *C. baccata* previously reported [19]. GC possesses a similar reducing power to the ones obtained for two brown seaweed, *S. muticum* [65] and *S. polyschides.* [20]. Finally, CC possesses the lowest reducing power of the red algae, being in the same range as the values obtained for the green seaweed *Ulva intestinalis* [21] and *Ulva lactuca* [22]. Similar results were obtained by Takei et al. when they analyzed the antioxidant activity of the aqueous extract solutions of eleven dried algal products, observing that the reducing power was higher in brown and red seaweed than in green seaweed [66]. Unfortunately, the current lack of an official method makes the comparison with data from the literature a difficult task. To illustrate this point, it could be mentioned that there are a few reports that express the reducing power of CC extracts as absorbance at 700 nm [67,68]. Among the studies that report the results using AAE/g algae as units, there are significant differences, due to both the extraction procedure employed and the analytical protocol used [69,70]. Contradictory results can also be found; for instance, there are studies that show aqueous extracts with higher reducing activity than ethanolic extracts [71], while several others claim the opposite. Regarding GC, to the best of our knowledge, this is the first report on the reducing activity of an aqueous extract of GC, since only studies with other *Gelidium* species were found in the literature [69]. It should be noted that, among the results obtained, the relation that was observed between GC and CC was the same as that observed between *Gelidium amansii* and CC [68]. Concerning PL, as happened with GC, previous studies on the reducing power of *P. linearis* were not found, but there are a few studies on different *Porphyra Spp* [67]. It has been observed that not all the *Porphyra* species possess the high reducing power observed in PL extract.

There are different studies dealing with the phenolic content of CC, but the results differ significantly from one another [72]. A few studies have reported on a similar value to the one obtained in this work [67,70], while others have found either significantly higher [68] or surprisingly lower values [73]. Finally, one study showed that CC aqueous extract presented the lowest phenolic content of the macroalgae analyzed [74].

Regarding the total phenolic content of GC, the value obtained in this study showed low TPC, with similar results to those obtained for the green seaweed previously mentioned. For the sake of comparison, TPC of different *Gelidium Spp* were consulted, but the results obtained were significantly different from one another, showing either higher or lower TPC for the same species [68,69,73].

In the case of PL extract, as expected from the results of the reducing power assay, the TPC was the highest among the red macroalgae studied, with a value of 1.83 ± 0.08 mg GAE/g seaweed. Different values had been provided by a previous study for a methanol and a dichloromethane extract of PL, where it could be observed how the TPC is affected by the solvent used [75]. Additionally, higher TPC had been observed for other species of *Porphyra* [67,72].

The results obtained in DPPH assays are also affected in a relevant way by the solvent used, as Farvin et al. demonstrated for ethanol and water extracts [67]. Furthermore, the results found in the literature are contradictory. For instance, two studies reported that methanol extract, acetone, and water extracts of CC did not possess DPPH scavenging activity [72,74], while another study reported a 20% scavenging activity for an ethanol extract at a concentration of 10 mg/mL [73]. In the same way, contradictory results were also observed for other species of *Gelidium.* Some works provided the results as percentage of scavenging activity at a fixed concentration [73], while it was claimed in another study that scavenging activity had not been found [68]. As expected, PL showed the lower IC50 value when compared with the other two red seaweeds. There are studies where even lower values were obtained when using methanol or dichloromethane extracts of PL [75]. However, for other species of *Porphyra,* higher IC50 values for *P. umbilicalis* [72] and *P. purpurea* were obtained in other works [67].

When comparing the results obtained for CC extract and Au@CC (Figure 8), in the case of the reducing power, a slight decrease in the value obtained for Au@CC could be observed. Regarding TPC, a significant decrease was observed in Au@CC, possessing half the phenolic content than before the synthesis. This implies that the phenolic compounds in CC extract actively contribute to the reduction process that takes place during the synthesis of Au@CC. In the case of the DPPH, a direct relationship with the reducing power could be observed, since a difference in behavior was observed, with a significant increase in the IC50 value in Au@CC. In the case of the Au@GC, when compared with GC extract, the results obtained showed a reduction by half in the reducing power in Au@GC. A reduction by half in the total phenolic content could also be observed in Au@GC after the synthesis of the NPs, which suggests its involvement in the reduction of the gold salt. Although significant differences could be seen in the reducing power and in the TPC, there were no significant differences in the case of the DPPH scavenging activity after the synthesis of Au@GC. Finally, in the case of PL extract and Au@PL, no significant difference was observed in the reducing power after the synthesis of the NPs. On the other hand, a decrease in the phenolic content was observed, which suggests, as happened with CC extract, the participation of the phenolic compounds from PL extract in the reduction process. Additionally, an increase in the DPPH scavenging activity was observed.

### 2.3. Evaluation of the Biological Activity In Vitro

#### 2.3.1. Antitumoral Activity in a Human Lung Epithelial and a Monocytic Cell Line

The potential antitumoral activity of Au@CC, Au@GC, Au@PL and their respective extracts was tested in two different cell lines, A549 lung epithelial and THP-1 monocytic cell line, by flow cytometry. The cells were labelled with Annexin V-FITC and PI to characterize the type of cell death induced by the samples (apoptosis, necrosis, or a mixed pattern).

Both the extracts and the NPs were tested at the same extract concentration (50 mg/mL) to determine any relevant differences between the three red macroalgae, except for Au@PL, in which case the extract concentration was lower (4 mg/mL) due to the superior capacity of PL to reduce Au(III) to Au(0), as explained in Section 2.1. For the NPs, the gold concentration is given in μM.

**Figure 9:**
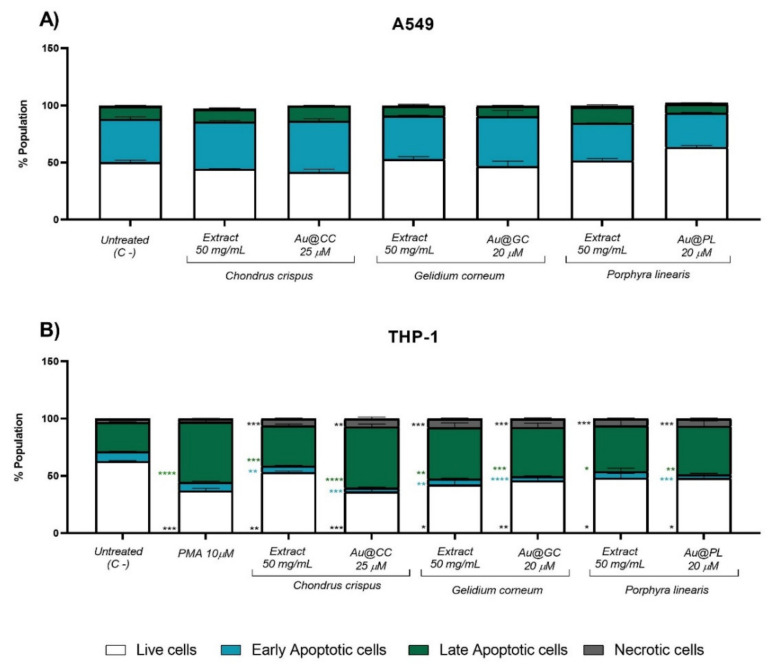
Percentage of live, early apoptotic, late apoptotic, and necrotic populations in (**A**) A549 and (**B**) THP-1 cell lines after 72 h of incubation with Au@CC, Au@GC, and Au@PL as well as the extracts used for the synthesis of the NPs. Cells incubated with PMA at 10 μM were used as positive control in the THP-1 cell line. * *p* ≤ 0.05, ** *p* ≤ 0.01, *** *p* ≤ 0.001, **** *p* ≤ 0.0001.

Figure 9 shows the different cellular populations induced by the NPs and the extracts in A549 (A) and THP-1 cells (B). After 72 h of incubation, no significant differences between A549 cells and untreated cells were observed in any of the AuNPs or the respective extracts at the concentrations tested. However, a significant increase mainly in the late apoptotic, but also in the necrotic cell population, was observed in THP-1 cells with the AuNPs and the extracts that was higher for the AuNPs. Interestingly, no relevant differences were found between CC, GC, and PL. Yet, in Au@PL the extract concentration was only 4 mg/mL compared to 50 mg/mL in Au@CC or Au@GC. Moreover, the cytotoxicity was mainly mediated by cell apoptosis in all the samples and not associated to inflammation, as in the case of necrosis [76].

This selective antitumoral effect on the monocytic cell line but not on the pulmonary epithelial cells was also described for AuNPs synthesized from a *Mastocarpus stellatus* carrageean extract at 15 μM [63]. Interestingly, twice as low a concentration also led to a significant apoptosis in the cells. Similarly, AuNPs synthesized from other red algae extracts, such as *Corallina officinalis*, showed a significant antitumoral effect on a breast epithelial cell line (MCF-7) [77,78]. However, the cytotoxicity was mediated by necrosis instead of apoptosis.

#### 2.3.2. Antioxidant Activity in Human Promyelocytic Cells

To characterize the antioxidant activity of the samples in a biological system, ROS release inhibition was tested in HL-60 (human promyelocytic) cells by flow cytometry, using a ROS fluorescent marker to label the cells.

All samples were tested at two different concentrations (see Materials and Methods section) and in the absence or in the presence of PMA, a known ROS inductor. PMA alone was used as positive control. After 6 h of incubation with the AuNPs and their respective extracts, the median fluorescence intensity (MFI) was quantified and compared to the negative control (untreated cells).

**Figure 10:**
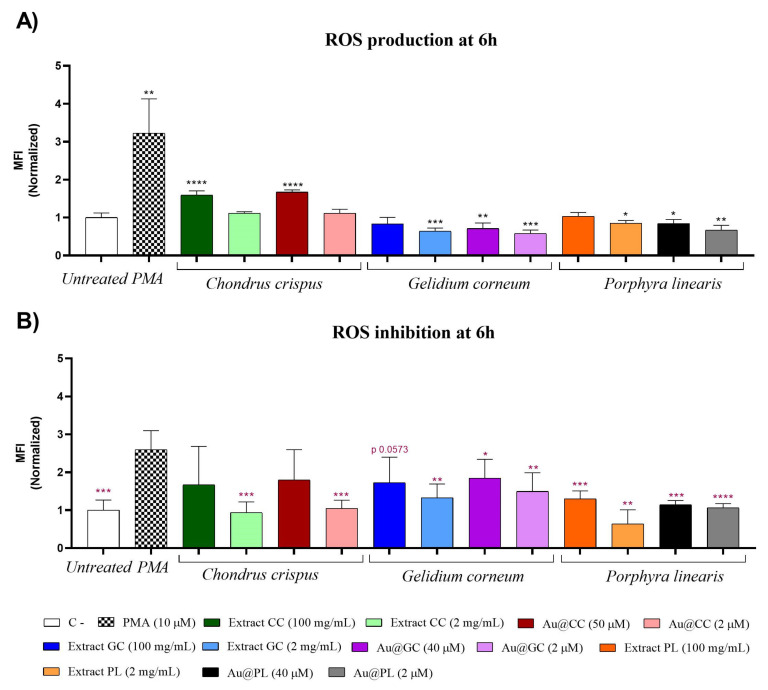
Median fluorescence intensity (MFI) of non-stimulated (**A**) or PMA-stimulated (**B**) HL-60 cells incubated for 6 h with the samples at two different concentrations and labeled with a ROS fluorescent marker. PMA alone was used as positive control. * *p* ≤ 0.05, ** *p* ≤ 0.01, *** *p* ≤ 0.001, **** *p* ≤ 0.0001.

Figure 10A shows the MFI of the cells incubated with the AuNPs and their respective extracts, i.e., in the absence of PMA. Interestingly, all the samples were able to reduce the basal ROS release, except for Au@CC and CC extract at both concentrations and PL at the highest concentration tested. In fact, Au@CC and CC extract at the highest concentration (100 mg/mL for the extract and 50 μM for Au@CC) led to a significant ROS increase in comparison with the untreated cells (Figure 10A) and no ROS inhibition was observed in the presence of PMA (Figure 10B). However, at low concentration, they achieved the highest ROS inhibition in cells pretreated with PMA (Figure 10B), together with Au@PL and PL extract at both concentrations tested. Moreover, their inhibitory effect was superior to that induced by Au@GC and GC extract. As regards the DPPH scavenging activity, Au@PL showed the highest antioxidant activity because it was able to reduce the ROS to basal levels at a lower extract concentration than the rest of the samples (8 mg/mL in Au@PL at 40 μM and 0.4 mg/mL in Au@PL at 2 μM). On the contrary, Au@GC induced a highest inhibition than its respective extract at the same concentration, while no relevant differences could be noted between Au@CC and its respective extract.

The dual effect in ROS production induced by Au@CC was also described for AuNPs synthesized from *U. intestinalis* and *S. polyschides* [20,21]. While at low concentration the extract induced an inhibitory effect, at high concentration the extract was able to increase basal ROS release. This dual effect could be useful in the regulation of some cellular processes that are mediated by ROS signaling [79].

In brief, all the red algae extracts showed antioxidant properties in the cells, which were improved by the synthesis of AuNPs, with the exception of CC at high concentration, as also described for AuNPs synthesized from a carrageenan extract from *M. stellatus* [63]. Similarly, AuNPs synthesized from non-algae extracts, such as *Brassica rapa* or chitosan, showed an increased antioxidant activity compared to the extract alone [80,81]. Interestingly, the shape of the NPs was also relevant to the antioxidant activity, being spherical NPs more efficient than irregular or polygonal ones [81]. The increased antioxidant activity of the NPs compared to the extracts could be associated with an improved internalization or a higher accumulation of the antioxidant molecules in the cells mediated by the NPs.

#### 2.3.3. Immunomodulatory Activity in a Cell Model of Inflammation

In addition to the antioxidant activity, CC, GC, and PL were also described to have anti-inflammatory or immunomodulatory potential [32,39]. Hence, to test the immunomodulatory effect of the AuNPs synthesized from the red algae extracts in a model of cell inflammation, some inflammatory cytokines were measured. Particular attention was paid to the potential synergistic or antagonistic effect of Au@CC, Au@GC, and Au@PL on the concentration of the chemokine MCP-1 and the proinflammatory cytokines IL-1β, IL-6, and TNFα induced by LPS in the monocytic cell line THP-1. Moreover, the extracts alone were also tested at the same concentration as in the NPs for comparison.

**Figure 11:**
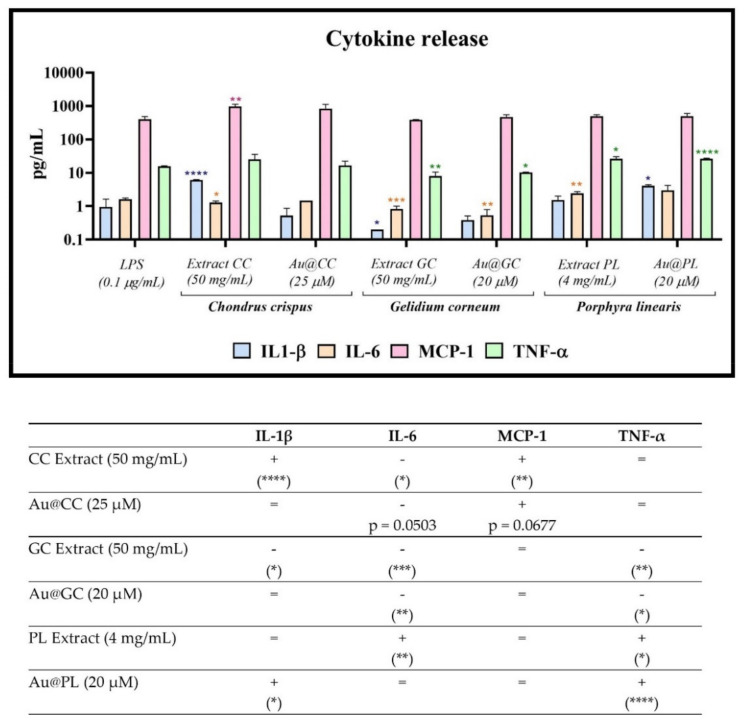
Cytokine concentration in THP-1 cells prestimulated with LPS at 0.1 μg/mL and incubated with the samples at two different concentrations (upper graph) and summary table of cytokine increase or inhibition induced by Au@CC, Au@GC, Au@PL, and their respective extracts. Cytokine concentration with respect to cells treated with LPS alone: = (equal); − (lower); + (higher). * *p* ≤ 0.05, ** *p* ≤ 0.01, *** *p* ≤ 0.001, **** *p* ≤ 0.0001.

Figure 11 shows the concentration of the selected cytokines in the cells treated with the extracts and the AuNPs (upper graph). The up or down regulation of the different proinflammatory cytokines compared to the positive control, LPS 0.1 μg/mL, was summarized in the table (lower data).

Only Au@GC was able to inhibit IL-6 and TNFα levels significantly compared to the positive control cells. Au@CC also inhibited the production of IL-6, but the reduction was not significant (*p* = 0.0503). In both cases, the NPs showed a reduced anti-inflammatory potential compared to that of the extracts. Moreover, GC extract induced a significant decrease in the level of IL-1β that was not observed after the synthesis of Au@GC. On the contrary, CC was able to induce an increase in this last cytokine that was not observed after the synthesis of Au@CC. Similarly, Au@PL showed a different behavior from that of the extract alone, and it was able to induce a synergistic effect on the production of IL-1β and TNF-α. Hence, some of the immunomodulatory molecules that were present in the aqueous extract could have been consumed or modified during the synthesis of the NPs.

Taking into account the capacity of AuNPs to regulate these inflammatory cytokines, Au@GC could be an alternative therapy for the treatment of inflammatory and autoimmune diseases mediated by a high concentration of IL-6 and/or TNFα, such as rheumatoid arthritis, inflammatory bowel disease, or cytokine storm [82,83]. Moreover, the immunomodulatory behavior of the NPs could prevent the increased risk of infection induced by conventional anti-IL6 and anti-TNFα therapies, associated with a down-regulation of these relevant cytokines [84,85].

Au@PL on its part could be useful as an adjuvant for vaccine development mediated by the release of TNFα and IL-1β, although its capacity to induce the release of these and other relevant cytokines involved in the immune response should be tested in non-stimulated cells. TNFα and IL-1β are relevant to the induction of an adaptive immune response as shown for other adjuvants, such as some lipopolysaccharides, zymosan, or inulin [86].

Moreover, TNFα is also involved in hematopoietic stem cell survival and myeloid differentiation, playing an essential role in tissue regeneration [87]. Hence, Au@PL could be useful in immunostimulant therapies where TNFα or IL-1β play an essential role.

The antioxidant and immunomodulatory properties of the bioactive compounds of the extracts, or the AuNPs, could be mediated by their interaction with the p38, JNK, NFkB, or the IRF3 signalling pathways, based on those mechanism activated by PMA (ROS stimulus) and LPS (inflammatory stimulus) [88,89]. However, the elucidation of the specific mechanisms involved in each biological effect was not the aim of this comparative study, and it would need further molecular biology studies.

## 3. Materials and Methods

### 3.1. Preparation and Characterization of Algae Extracts

Thalli of live CC, GC and PL were collected at the lower intertidal rocky shore on the NW coast of Spain (42°12′2.9″ N; 8°47′6.2″ W). For the sake of comparison, the aqueous extracts of the three algae were prepared following the procedure previously reported [19].

Briefly, to obtain the aqueous extracts the seaweeds were thoroughly rinsed to remove seawater, sand, and associated biota and then were cut into fine pieces and treated in boiling water for 15 min. The extract was centrifuged at 4500 rpm for 10 min, and the supernatant was filtered.

During this treatment, polar compounds are preferentially extracted from algae. As in previous works, we evaluated the presence of various carbohydrates that could be the main responsible for the reduction of Au(III) to Au(0).

### 3.2. Analysis of Carbohydrates

The characterization of the carbohydrate fraction was performed by size exclusion chromatography, by means of an Agilent 1200 Series system (Agilent Technologies, Palo Alto, CA, USA), using a PL aquagel-OH 40 column (particle size 8 μm, 300 × 7.5 mm). The liquid chromatograph was coupled to an Agilent 1260 Infinity refractive index detector. The elution was achieved isocratically, using a mobile phase containing water and a small percentage of sodium azide (0.2%), at a flow of 0.5 mL/min and at room temperature. The injection volume was 100 μL.

The pre-treatment of the samples was a simple centrifugation and a filtration of the aqueous extracts, using filters with a pore size of 0.2 μm. Prior to the analysis, the solutions were purified using a Dionex OnGuard II P cartridge with the aim of removing potentially interfering compounds such as polyphenols.

A mixture of dextrans with molecular weights in the range from 150 to 12 kDa (from Sigma Aldrich, San Luis, MO, USA) was used as a reference standard for calibration. The standard solutions were analyzed under the same eluting conditions, and their retention times were recorded and marked on chromatograms. All analyses were performed by triplicate.

### 3.3. Synthesis of AuNPs

The optimal reaction conditions for the synthesis of homogeneous AuNPs employing CC extract (Au@CC), GC extract (Au@GC), and PL extract (Au@PL) were determined after several trials. Different ratios of seaweed extract and gold salt were tested, and, in all cases, the reaction outcome was monitored by color change and UV-Vis spectroscopy. Briefly, different volumes of HAuCl_4_ 0.01 M were slowly added to the seaweed extract, and the solution was kept at room temperature (RT) while stirring until color changed. The best reaction conditions are shown in the Results and Discussion section.

### 3.4. Characterization of AuNPs

A Jasco Spectrometer V-670 was used for the acquisition of UV-Vis spectra at room temperature. The concentration of gold was determined by using a Perkin Elmer Optima-4300 DV ICP-OES with Indium as internal standard.

Zeta potential of Au@CC, Au@GC, and Au@PL were obtained through electrophoretic mobility by taking the average of five measurements at the stationary level using a ZetasizerNano S (Malvern Instruments, Malvern, U.K.) equipped with 4 mW He−Ne laser, operating at a wavelength of 633 nm.

Samples for Fourier Transform Infrared Spectroscopic Analysis (FTIR) were prepared by placing the extracts and the NPs solutions in an oven at 80 °C until they dried. The dried materials were ground to fine powder and used to record the spectra by means of KBr pellet technique. FTIR spectra of the extracts and their NPs were recorded by using a Jasco FT/IR-6100 spectrophotometer in the range of 4000–400 cm^−1^ at a resolution of 4 cm^−1^. The FTIR spectra of commercial carrageenan and agar were recorded for comparative purposes.

Au@CC, Au@GC, and Au@PL samples for electron microscopy characterization were centrifuged at 10,000 rpm for 30 min to eliminate part of the extract. Then, the pellets were dispersed in milliQ water and sonicated for 15 min. Finally, a drop of the dispersion was placed onto holey carbon film on a copper grid. Transmission Electron Microscopy (TEM) images were acquired with a JEOL JEM 1010 (100 kV), while High-Resolution Transmission Microscopy (HRTEM) and Scanning Transmission Electron Microscopy (STEM) images were acquired with a JEOL JEM2010F field emission gun TEM, operated at 200 kV or with a JEOL JEM-2200FS (200 kV). Electron Energy Loss Spectroscopy (EELS) measurements were performed in STEM mode using a Gatan Quantum EELS GIF, with a collection semi-angle of β = 16.75 mrad. Energy resolution was ~1.75 eV (FWHM of the zero-loss peak). To avoid the contribution of the carbon film, EELS spectra were measured in areas of the sample positioned over a hole. Coupling between the STEM unit and the EDS detector (Oxford Inca Energy 200) was used to obtain elemental maps. Data collection and analyses were carried out using Digital Micrograph software by Gatan.

### 3.5. Evaluation of the Antioxidant Activity

The antioxidant and antiradical activity of CC, GC, and PL extracts before and after the synthesis of AuNPs were analyzed by means of three assays: DPPH radical scavenging activity, reducing power, and total content of phenols. The determination was performed following the protocols previously reported [19,90].

### 3.6. Evaluation of the Biological Activity In Vitro

A549 (lung epithelial), THP-1 (monocytic), and HL-60 (promyelocytic) cell lines, provided by the American Type Culture Collection (ATCC), were selected as human cell models. These cells were cultured according to the ATCC recommendations.

Unless otherwise stated, the samples were tested at 50 mg/mL for all extracts, 25 μM (Au) (50 mg/mL extract) for Au@CC, and 20 μM (Au) (50 mg/mL and 4 mg/mL extract) for Au@GC and Au@PL, respectively.

#### 3.6.1. Apoptotic Assay

Cellular apoptosis/necrosis induced by the AuNPs and the extracts in two different cell lines, A549 and THP-1, were studied by flow cytometry using a double labelling with Annexin V-FITC and Propidium Iodide (PI).

A549 and THP-1 were seeded at a density of 7 × 10^3^ cells/well and 1 × 10^4^ cells/well, respectively, and allowed to rest overnight. After that, the samples were added. Culture medium alone was used as a negative control while phorbol 12-myristate-13-acetate (PMA) at 10 μM was employed as a positive control for THP-1 cells.

After 72 h of incubation, the cells were washed with PBS, labeled with Annexin V-FITC and PI, following the manufacturer’s instructions, and analyzed by flow cytometry (Beckman Coulter FC500, Brea, California, USA).

#### 3.6.2. Evaluation of the Antioxidant Activity in Cells

The potential inhibition of ROS release by the samples was evaluated in HL-60 cells as described before in Diego-González et al., 2020 [91]. Two different concentrations were tested for each sample to characterize any dose-dependent effects: 100 and 2 mg/mL for all extracts; 50 μM (Au) (100 mg/mL extract) and 2 μM (Au) (4 mg/mL extract) for Au@CC; 40 μM (Au) (100 mg/mL extract) and 2 μM (Au) (5 mg/mL extract) for Au@GC; and 40 μM (Au) (8 mg/mL extract) and 2 μM (Au) (0.,4 mg/mL extract) for Au@PL.

#### 3.6.3. Cell Model of Inflammation and Cytokine Release

The human monocytic cell line THP-1 stimulated with lipopolysaccharide (LPS) was used as a model of cell inflammation. Cells were seeded at a density of 1.5 × 10^4^ cells/well in a 96-well plate and allowed to rest for 24 h. Then, LPS at 0.1 μg/mL was added to the cells 3 h before the addition of the treatments. Culture medium alone and LPS at 0.1 μg/mL were used as negative and positive controls, respectively. After 24 h of incubation, the plates were centrifuged, and the supernatants were collected for cytokine measurements.

A MILLIPLEX^®^ MAP Kit for Monocyte Chemoattractant Protein-1 (MCP-1), Tumor Necrosis Factor Alpha (TNF-α), Interleukin 1 Beta (IL-1β), and Interleukin 6 (IL-6) was used to measure the concentration of these cytokines in the supernatants using a MAGPIX^®^ instrument (Millipore, Merck KGaA, Darmstadt, Germany) and following the manufacturer’s instructions. MAGPIX^®^ software (V4.2, Millipore, Merck KGaA, Darmstadt, Germany) was used for the analysis of the standard curves and the samples.

### 3.7. Statistical Analysis

After determining the distribution of the samples by a Shapiro–Wilk test, a T-student or Mann–Whitney test was conducted to ascertain significant differences between control and treatments using GraphPad Prism 8 software. The results were represented as mean ± standard deviation (SD), and, in the graphs, statistically significant results are referred to as: * *p* ≤ 0.05, ** *p* ≤ 0.01, *** *p* ≤ 0.001, **** *p* ≤ 0.0001.

## 4. Conclusions

A comparative study of the synthesis of AuNPs led by aqueous extracts from three different red macroalgae has been presented. The full characterization performed confirmed the formation of spherical, polycrystalline NPs, with mean sizes of 16.9 ± 2.5, 15.0 ± 3.0, and 44.2 ± 6.1 nm for Au@CC, Au@GC, and Au@PL, respectively. In all cases, as shown by Z potential measurements, the NPs carry negative charge, possibly due to the polysaccharides present in the samples. In this regard, the HPSC and FTIR analyses suggest not only that polysaccharides are involved in the synthesis and stabilization of the NPs but also the participation of other biomolecules such as proteins and poliphenols.

In general, AuNPs showed an improved antitumoral and antioxidant activity in comparison with that of the extract. In particular, Au@CC, Au@GC, Au@PL induced a significant antitumoral effect in a monocytic cell line, mediated by cell apoptosis. All of them presented a relevant antioxidant activity, but Au@PL induced the highest antioxidant activity in cells exposed to oxidative stress at low and high concentrations, in agreement with the results obtained in the DPPH scavenging assays, while Au@CC showed a dual effect on ROS production that was dose-dependent.

Au@GC and Au@PL displayed an intrinsic immunomodulatory activity, which could be further explored for immunotherapeutic applications. For instance, Au@GC could be an alternative therapy in the treatment of chronic inflammatory diseases mediated by an increased concentration of IL-6 and TNFα, such as rheumatoid diseases, on the basis of the capacity of the NPs to inhibit these cytokines. The immunomodulatory capacity of the NPs could avoid the side-effects associated to an inhibitory therapy, e.g., the use of antibodies or cytokine receptor antagonists. Besides, Au@PL induced the synergistic release of TNFα and IL-1β, two cytokines that are valuable in immunostimulant therapies such as vaccination.

## Figures and Tables

**Table 1:** Optimal reaction conditions for the synthesis of AuNPs from three different red algae extracts.

Algae	[Extract] (g/mL)	[Au] (mM)	T (°C)	t (h)	Code
*C. crispus*	1	0.5	30	48	Au@CC
*G. corneum*	1	0.4	30	24	Au@GC
*P. linearis*	0.08	0.4	30	24	Au@PL

## Data Availability

The data used to support the findings of this study are included within the article.
